# Predictive Modelling of Susceptibility to Substance Abuse, Mortality and Drug-Drug Interactions in Opioid Patients

**DOI:** 10.3389/frai.2021.742723

**Published:** 2021-12-10

**Authors:** Ramya Vunikili, Benjamin S. Glicksberg, Kipp W. Johnson, Joel T. Dudley, Lakshminarayanan Subramanian, Khader Shameer

**Affiliations:** ^1^ Department of Computer Science, Courant Institute of Mathematical Sciences, New York University, New York, NY, United States; ^2^ Department of Information Services, Center for Research Informatics and Innovation, Northwell Health, New York, NY, United States; ^3^ Bakar Computational Health Sciences Institute, University of California, San Francisco, San Francisco, CA, United States; ^4^ Institute for Next Generation Healthcare, Mount Sinai Health System, New York, NY, United States

**Keywords:** addicition, opiod abuse, digital health, predictive modeling, machine learing

## Abstract

**Objective:** Opioids are a class of drugs that are known for their use as pain relievers. They bind to opioid receptors on nerve cells in the brain and the nervous system to mitigate pain. Addiction is one of the chronic and primary adverse events of prolonged usage of opioids. They may also cause psychological disorders, muscle pain, depression, anxiety attacks etc. In this study, we present a collection of predictive models to identify patients at risk of opioid abuse and mortality by using their prescription histories. Also, we discover particularly threatening drug-drug interactions in the context of opioid usage.

**Methods and Materials:** Using a publicly available dataset from MIMIC-III, two models were trained, Logistic Regression with L2 regularization (baseline) and Extreme Gradient Boosting (enhanced model), to classify the patients of interest into two categories based on their susceptibility to opioid abuse. We’ve also used K-Means clustering, an unsupervised algorithm, to explore drug-drug interactions that might be of concern.

**Results:** The baseline model for classifying patients susceptible to opioid abuse has an F1 score of 76.64% (accuracy 77.16%) while the enhanced model has an F1 score of 94.45% (accuracy 94.35%). These models can be used as a preliminary step towards inferring the causal effect of opioid usage and can help monitor the prescription practices to minimize the opioid abuse.

**Discussion and Conclusion:** Results suggest that the enhanced model provides a promising approach in preemptive identification of patients at risk for opioid abuse. By discovering and correlating the patterns contributing to opioid overdose or abuse among a variety of patients, machine learning models can be used as an efficient tool to help uncover the existing gaps and/or fraudulent practices in prescription writing. To quote an example of one such incidental finding, our study discovered that insulin might possibly be interacting with opioids in an unfavourable way leading to complications in diabetic patients. This indicates that diabetic patients under long term opioid usage might need to take increased amounts of insulin to make it more effective. This observation backs up prior research studies done on a similar aspect. To increase the translational value of our work, the predictive models and the associated software code are made available under the MIT License.

## Introduction

Drug overdose is the leading cause of accidental deaths in the US, with 52,404 lethal drug overdoses in 2015 (Rudd et al., 2016). Opioid use disorder is the primary driver of the epidemic, with 20,101 overdose deaths related to prescription pain relievers and 12,990 overdose deaths related to heroin in 2015 (Rudd et al., 2016). This has become known in popular culture as the “Opioid Epidemic.” The overdose death rate in 2008 was nearly four times that in 1999 and the sales of prescription pain relievers in 2010 were four times those in 1999 (Hall et al., 2008). A study done by Jeffery et al., highlights the fact that despite all the increased attention to opioid abuse and awareness of risks, the opioid use and average daily dose have not substantially decreased from their peaks (Jeffery et al., 2018). Drug overdose continues to be an alarming public health problem and thus, it needs immediate attention. However, a part of this problem could be addressed if we can pre-emptively identify those patients who are most susceptible to adverse outcomes when prescribed opioid or opiate therapies. We provide a potential solution to this by using simple yet robust machine learning techniques involving classification algorithms. In addition to this identification task, we’ve also explored the interactions between opioids and other drugs that could result in increased incidence of side effects by performing a K-Means clustering. This exercise acts as a testimony for the ability of machine learning algorithms to look at complex patterns efficiently and uncover the most relevant ones. Also, as aptly described in Khader et al., this study combines the robustness of both statistical analysis and machine learning techniques (Shameer et al., 2018) and also exemplifies the utility of publicly available biomedical datasets and its application for improving public health as emphasized by Khader et al (Shameer et al., 2017). Despite its status as a major problem in American healthcare, the opioid epidemic has been understudies by artificial intelligence researchers who work on problems in healthcare. The study done by Che et al., is one of the few attempts to classify patients based on opioid usage (Che et al., 2017). This study categorizes patients into three groups (short term, long term and opioid dependent users) based on the number of prescriptions given.

Here, opioid dependent users refer to those who are diagnosed with “opioid dependence.” This study describes two classification tasks: a) whether a short-term user will turn into a long-term user and b) whether a long-term user is an opioid dependent user. One issue with such a type of classification is that the study is ignoring the possibility of a short-term user developing the symptoms of opioid dependence.

When a patient is prescribed opioids only a few times but with high dosages the patient could still be prone to adverse effects and/or drug-drug interactions (Bartoli and Kominek, 2019). We note that in this study, the best performing model for identifying opioid dependent users is a deep learning model that uses Recurrent Neural Network (RNN). As highlighted by Miotto et al., deep learning models perform better when trained on large datasets.8 However, as the number of patients who experienced opioid dependence symptoms in Che et al., was only 749, this study has randomly generated 14 datasets by downsampling non-opioid-dependent patients which formed two-thirds of the dataset and then trained the RNN model.6 This might not be the most technically robust way to generate data. Even with such a random generation the accuracy of the model is found to be 76.07% with a recall of only 52.05%. That means, the chances of identifying a long-term patient who could be prone to opioid dependence using this model is better than tossing a fair coin by a mere margin of 2%. Also, as Miotto et al., pointed out, deep learning models are often regarded as models lacking interpretability in healthcare (Miotto et al., 2017). To overcome all these issues, our study advocates the use of more interpretable machine learning models to achieve better classification accuracies by extracting data in a more robust way.

Another study done by Averill et al., was aimed at improving the decision-making in opioid-analgesic prescriptions through a model called Opioid Abuse Risk Screener (OARS) (Averill et al., 2017). OARS is a Support Vector Machine (SVM) based model and has performed better than the widely used Screener and Opioid Assessment for Patients with Pain Revised (SOAPP-R) both as a predictor of aberrant same-day urine drug testing (UDT) and aberrant controlled substance database (CSDB) checks within 1year of assessment date (Butler et al., 2008; Butler et al., 2009). A recent study done by Gong et al., used probabilistic modelling to identify phenotypes responsible pertinent to opioid use and opioid use disorders (Gong et al., 2018). These phenotypes were predictive of future opioid use-related outcomes. In addition to these, Wong et al. briefed about how Natural Language Processing (NLP) can effectively automate medication safety tasks and near real-time identification of adverse events for post-marketing surveillance (Wong et al., 2018).

Calcaterra et al., built a parsimonious statistical model for predicting hospitalized patients who will progress to chronic opioid therapy (COT) following their discharge from the hospital (Calcaterra et al., 2018). This model predicted COT correctly in 79% of the patients and no COT in 78% of the patients. Interestingly, a study done by Chiu et al., suggested that lowering the default number of opioid pills prescribed in an Electronic Medical Record (EMR) system can eventually change prescriber behavior and decrease the amount of opioid medication prescribed after procedures (Chiu et al., 2018). However, Steinman et al., explain the psychological obstacles involved in discontinuing a medication even if they’re found to be inappropriate (Steinman and Landefeld, 2018).

Apart from the above-mentioned preemptive methods which are still under active research, the healthcare sector is already using antagonists like naltrexone and naloxone as an alternative treatment to opioid addiction. Latif et al., conducted a randomized clinical trial in abstinence motivated adults with opioid dependence and assessed symptoms of anxiety, depression, and insomnia periodically. It was found that the Extended-Release Naltrexone and combined buprenorphine-naloxone worked equally well for anxiety and depression while the former gave a significantly lower score for insomnia (Latif et al., 2018).

Genotyping-based drug therapy decision could be another solution for this problem. Kringel et al., suggested separating pain patients requiring extremely high opioid doses from controls by using a bioinformatics-based classifying biomarker that uses emergent properties in genetics (Kringel et al., 2016).

In 2016, Center for Disease Control and Prevention (CDC) proposed a framework and guidelines for better and safer prescribing of opioids (Dowell et al., 2016). Furthermore, many researchers emphasized the role of education in restricting the opioid prescriptions. Tyndale et al., suggested that the prescribers and patients could change their behavior and benefit from being educated about pain management (Tyndale and Sellers, 2018). Wiese et al., also highlighted that not only postgraduate professionals but also pre-graduate health professionals require intensive integrated education efforts (Wiese et al., 2018).

## Materials and Methods

### Dataset

The MIMIC-III dataset is a publicly released, deanonymized dataset consisting of data from 46,520 patients at the Beth Israel Deaconess Medical Center, Boston, Massachusetts. Among these patients, 29,959 patients were identified with prescriptions of opioids or opiates such as Morphine, Meperidine, Codeine, Buprenorphine, Hydromorphone, Methadone, Fentanyl, Oxycodone, Oxymorphone, and Hydrocodone. Furthermore, 1,405 patients out of these were prescribed Naloxone, which is an anti-narcotic medication known for its usage as opioid overdose reversal drug. In a few cases, Buprenorphine could also be prescribed in combination with Naloxone to minimize the possibility of opioid dependence. In order to accommodate the fact that such opioids have mixed traits of triggering and treating opioid dependence, they are considered as both narcotic and anti-narcotic drugs simultaneously for this study.

### Cohort Selection

All the patients with opioid prescriptions are divided into eight age groups. Age is calculated by taking the difference of the date of birth of the patient and the date of prescription issued. The statistics of each of these age groups is presented in Table 1.

**TABLE 1:** Statistics of subjects in different age groups.

Age group	Age range (Years)	Total no. Of subjects	No. Of subjects with side effects	Proportion
1	<13	269	0	0.000
2	13–19	253	7	0.028
3	20–40	2949	254	0.114
4	41–50	3273	203	0.062
5	51–65	8507	251	0.030
6	66–75	5974	26	0.004
7	76–85	5906	7	0.001
8	>85	2861	1	0.0003

In order to create a better balance between the patients with side effects and those without side effects, the age boundaries of each group are adjusted such that the group has a good proportion of both these patients. This would help in choosing groups with higher proportion to be retained.

In order to identify patients with side effects, we checked the diagnoses of every patient prescribed with opioids for symptoms related to overdose and/or dependence using the International Classification of Diseases, Ninth Revision (ICD 9) codes. A few of the ICD nine codes and categories are listed in Table 2. A total of only 749 patients were identified to have side effects. This table is prepared based on the information released by the National Center for Biotechnology Information and Moore et al. (Heslin et al., 2015; Moore and Barrett, 2017) Also work done by Koob et al., suggested that psychostimulants can cause dependence in their works (Koob and Moal, 2006; Koob et al., 2014).

**TABLE 2:** List of ICD 9 codes used for identifying subjects with adverse events.

Broad category	ICD 9 codes
Opioid type or combination of opioid type with other drug dependence	30400 30401 30402 30403 30470 30471 30472 30473 30550 30551 30552 30553 96500 96501 96502 96509
Psychological effects	30410 30411 30412 30413 30540 30541 30542 30543
Psychostimulant dependence	30440 30441 30442 30443
Poisoning	96502 96509 9701 E8500 E8501 E8502
Hallucinogen dependence	30450 30451 30452 30453
Miscellaneous dependence	30420 30421 30422 30423 30430 30431 30432 30433

## Data Extraction Methodologies

### Feature Selection

**FIGURE 1:**
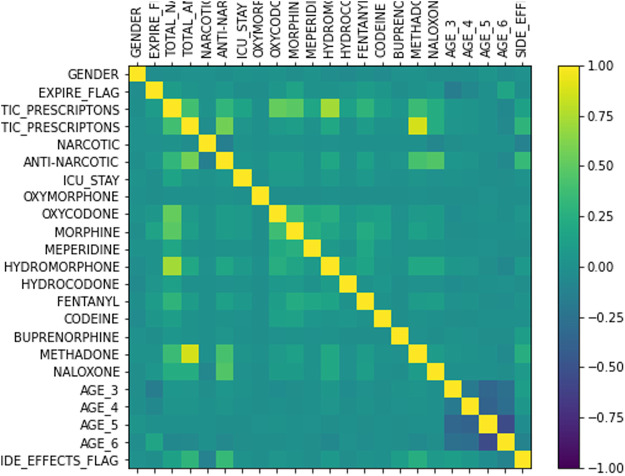
Correlation of features.

A total of 25 features are chosen using data-driven techniques to represent the opioid prescription information of the selected cohort. The target variable, SIDE EFFECTS FLAG, is set to 1 if the patient is diagnosed with any of the adverse events listed in Table 2 and 0 otherwise. The gender of a patient is represented by a binary variable - 0 for female and 1 for male. For patients with one or more Naloxone prescriptions, the ANTI NARCOTIC flag is set to 1 and for those with opioids of mixed traits both NARCOTIC and ANTI NARCOTIC are set to 1. For the prescriptions of all other opioids under study, the NARCOTIC flag is set to 1. Every opioid is allocated a discrete variable to represent the total number of prescriptions of that particular opioid given to each patient. In addition, the total number of anti-narcotic (Naloxone) and narcotic (opioids excluding Naloxone) prescriptions are represented by two discrete variables. If a patient had stayed in Intensive Care unit (ICU) then the binary flag, ICU, is set to 1 and 0 otherwise. Finally, feature normalization is done by performing an affine transformation on each feature so that all the values in the dataset are in the range of (0,1). Figure 1 shows the correlation of features. The target variable, SIDE EFFECTS FLAG, has the highest positive correlation with TOTAL ANTI NARCOTIC PRESCRIPTIONS and ANTI NARCOTIC flag. Intuitively, this makes sense because a patient would be treated with anti-narcotics when adverse events start to arise. Also, among opioids the number of prescriptions associated with BUPRENORPHINE and METHADONE have a relatively higher positive correlation with the target variable. In a few cases, the prescriptions of patients did not have the start and/or end dates. Such instances are dropped from the study. Also, another reasonable assumption made in the study is that the patients with a NAN value for ICU ID haven’t stayed in the ICU.

### Dealing With Class Imbalance

It can be observed from Table 1 that there is a large imbalance between patients with side effects and those without side effects. Running a classification algorithm on such a data would result in overfitting the model and hence it will learn to predict the majority class. As a result, the classification accuracy might be high even when the number of true positives for patients with SIDE EFFECTS FLAG as 1 is terribly low. This is evident from 3 which shows a huge difference between precision and recall.

We’ve taken the following two steps to address this problem:

• Down-sampling majority class

Among 749 patients identified with side effects, only 15 belonged to age groups 1, 2, 7, and 8. On the other hand, these age groups accounted for, approximately, 10,000 samples of majority class. Although excluding these age groups has resulted in a much better ratio of patients with side effects to those with no side effects (734:19969 vs. 749:29959), the data is still highly imbalanced.

• SMOTE—Oversampling minority class

In order to deal with the high class imbalance in the data, Synthetic Minority Oversampling Technique (SMOTE) developed by Bowyer et al., was used (Bowyer et al., 2011). This algorithm works by choosing the nearest neighbors of data with minority class label and upsamples them. This method was used after performing Linear Discriminant Analysis (LDA) on the data which provided evidence that both the classes were quite separable from each other. Implementing this algorithm not only led to the expansion of the dataset in a statistically robust way but also minimized the imbalance in the dataset.

## Principal Component Analysis for Addressing the Issue of Sparse Features

**FIGURE 2:**
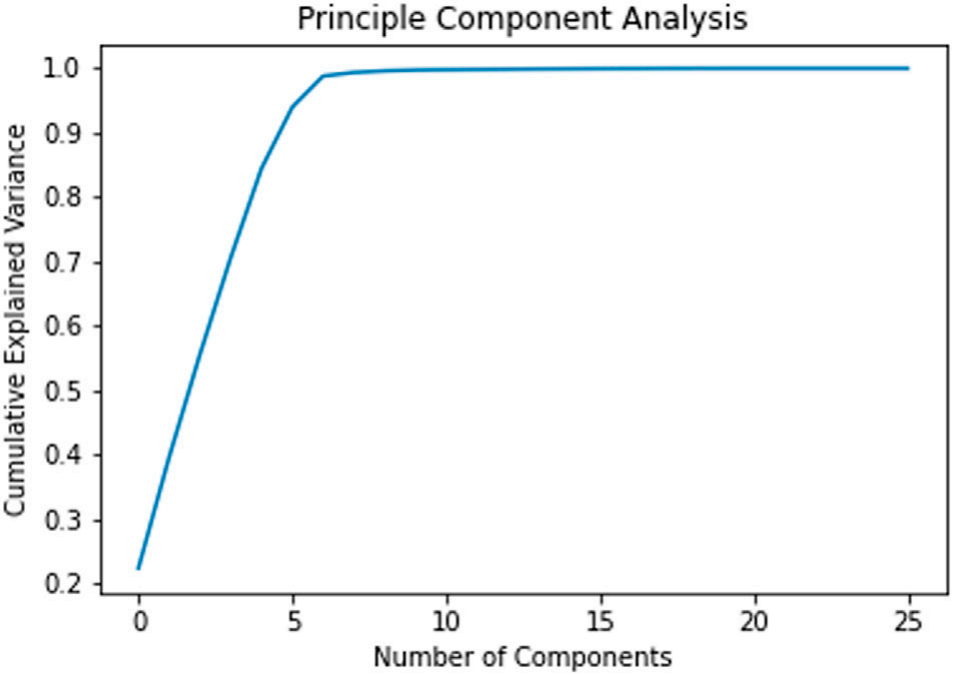
Cumulative explained variance across different principle components.

As described earlier, quite a number of features were based on the opioids given to the patients. A few opioids like Morphine were prescribed very often while the other opioids such as Oxymorphone were rarely prescribed. As every patient had features related to every opioid, the less frequently prescribed opioids led to sparse features. In order to have a better subset of features, we performed Principal Component Analysis. From Figure 2, it can be observed that the maximum variance is retained from component 6 onwards. But, the regression resulted in maximum accuracy with 11 components. Hence, the number of features have been reduced to 11.

## Modelling

The entire dataset was split into 80% training set and 20% test set for running the classification models. We’ve chosen Logistic Regression with L2 regularization as a baseline and Extreme Gradient Boosting (XGBoost) developed by Chen et al., as an enhanced model (Chen and Guestrin, 2016). For both the models, 20% of the training set was set aside as the validation set. Grid Search was done over this validation set to get the best parameters for the model.

### Baseline Model—Logistic Regression

**FIGURE 3:**
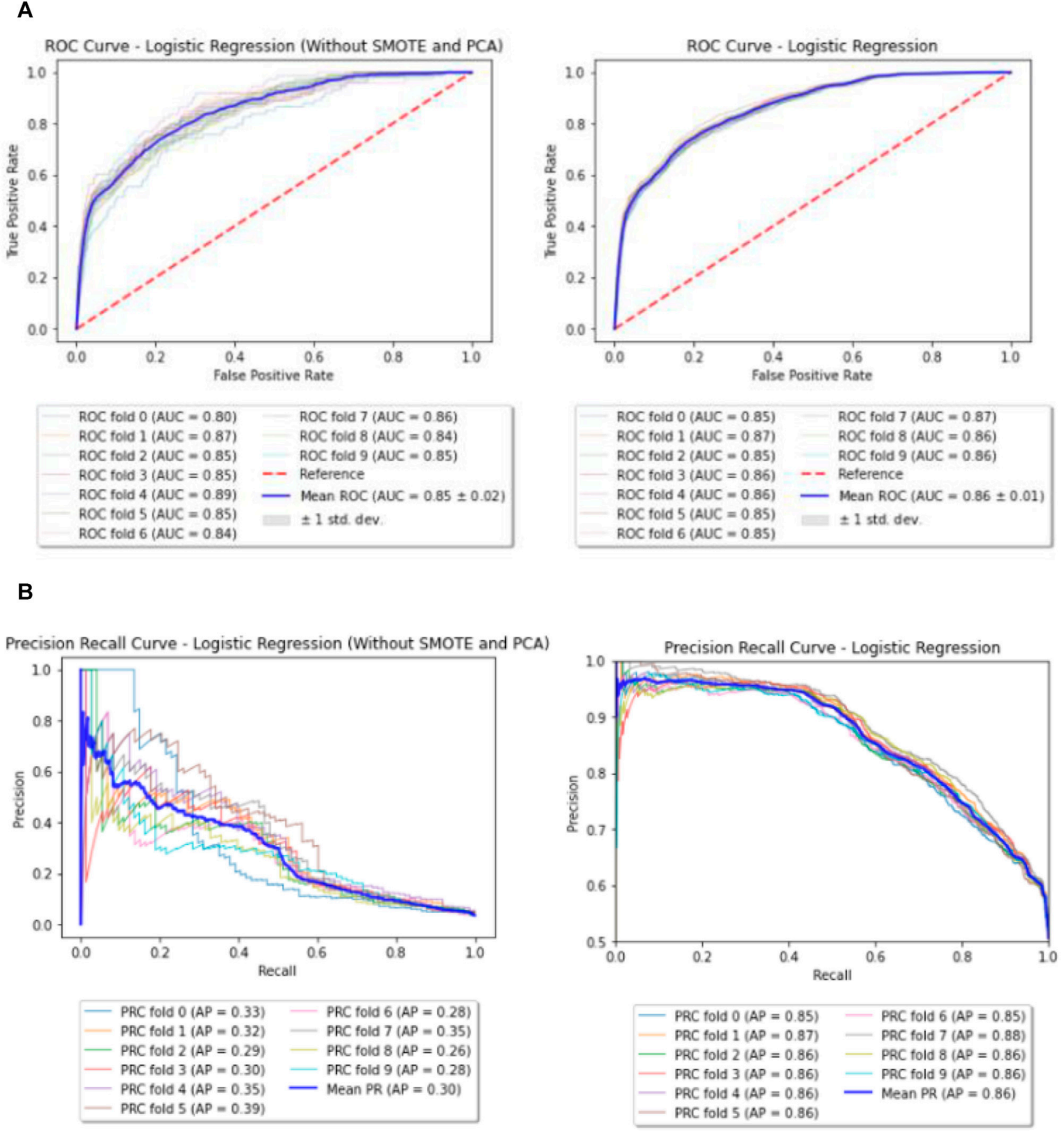
**(A)** Baseline—ROC curves (before and after performing SMOTE and PCA). **(B)** Baseline—Precision Recall (PR) curves (before and after performing SMOTE and PCA).

Logistic Regression model with L2 penalty of 0.001 was run on the dataset before and after performing SMOTE and PCA. The mean AUC and Precision Recall curves with average precision (AP) from 10 fold cross validation can be observed in Figures 3A,B.

### Enhanced Model—XGBoost

**FIGURE 4:**
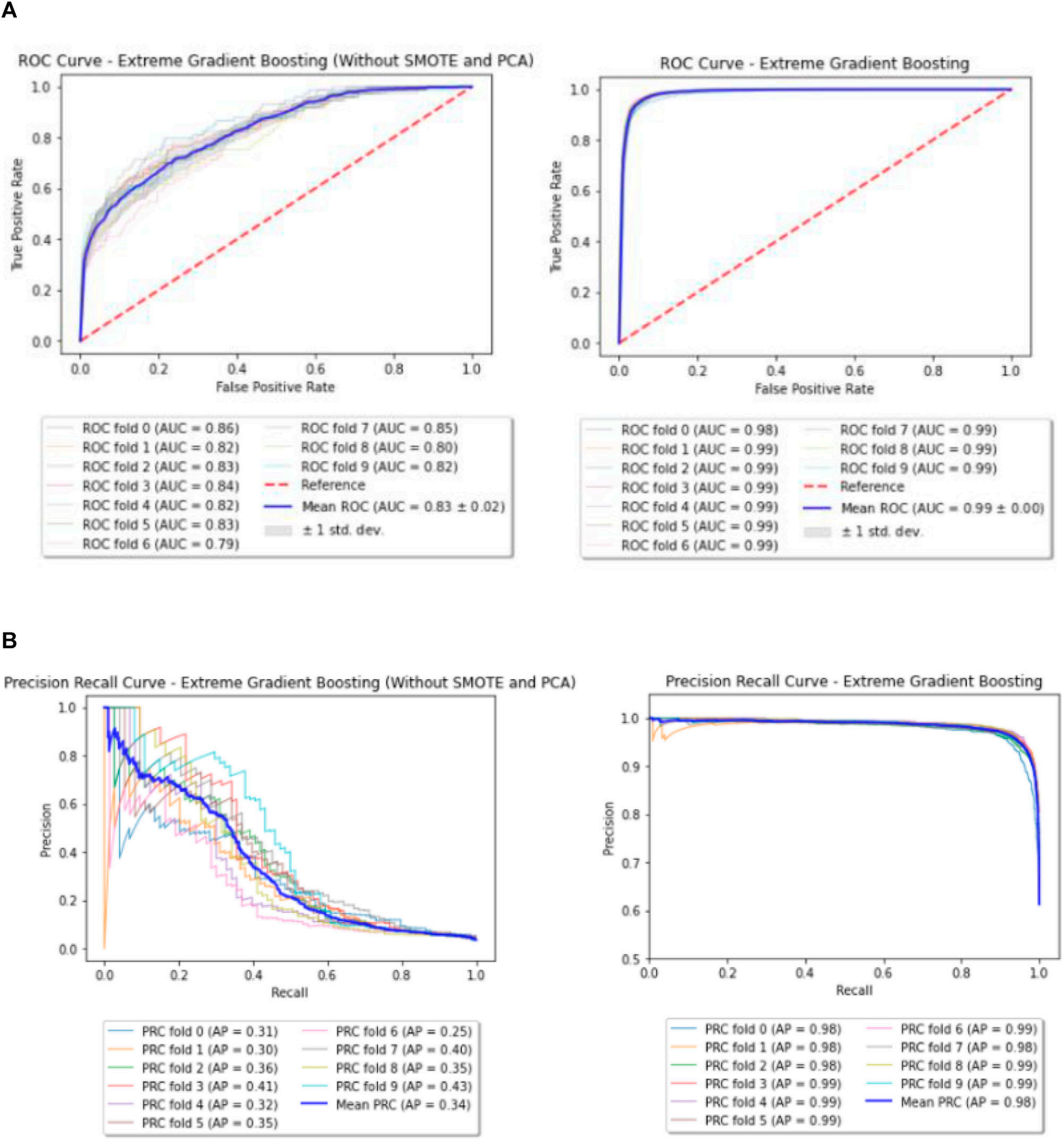
**(A)** Enhanced model—ROC curves (before and after performing SMOTE and PCA). **(B)** Enhanced model—Precision Recall (PR) curves (before and after performing SMOTE and PCA).

For XGBoost, the best parameters obtained through Grid Search are listed in Table 3. Few of the important parameters include max depth and reg lambda. While a higher max depth for each tree let’s the model capture interactions specific to a particular sample, reg lambda is similar to L2 regularization in the Logistic Regression. Both these parameters control over-fitting of the model and hence provide better performance over the Baseline. Also, the Receiver Operating Characteristic (ROC) curve and Precision Recall curve (PRC) before and after performing SMOTE and PCA for XGBoost are shown in Figures 4A,B.

**TABLE 3:** Summary of best parameters for XGBoost.

Parameter	Value
learning rate	0.1
max depth	10
n estimators	200
objective	“binary: logistic”
Base score	0.5
booster	“gbtree”
max delta step	0
colsample bylevel	1
colsample bynode	1
req alpha	0
req lambda	1
scale pos weight	1
gamma	0

### Mortality as the Target Variable

Until now we attempted to predict if a patient will show side effects when prescribed opioids. But a far more fatal consequence associated with opioids is loss of life. Being able to segregate patients with high risk of mortality could be a huge problem in itself. Therefore, to facilitate such a preemptive identification, we ran a classification algorithm on the cohort that has experienced side effects. XGBoost model was trained on 80% of these patients (*n* = 587) and tested on the remaining 20% (*n* = 147). The accuracy of the model is given in the Table 4.

**TABLE 4:** Summary of performance for predictive modeling tasks.

Model	Target variable	Precision (PPV)	Recall (%)	NPV (%)	F1 score(%)	Accuracy (%)
Logistic Regression	Side effects	78.45	74.91	75.60	76.64	77.17
XGBoost	Side effects	92.64	95.45	95.30	94.02	94.35
XGBoost	Mortality	66.67	31.82	76.20	43.07	74.83

### Interactions Between Opioids and Other Drugs

**FIGURE 5:**
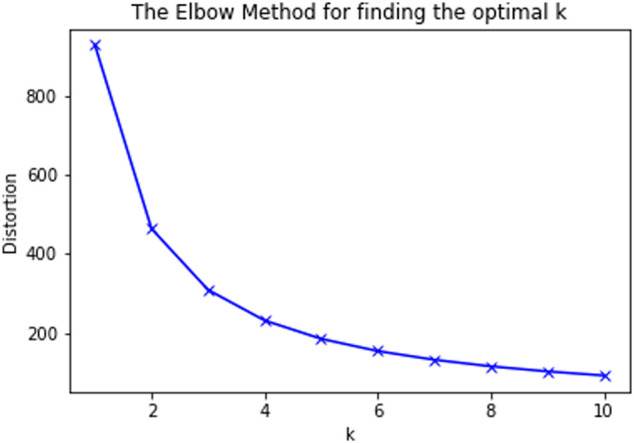
Elbow plot for K-means clustering.

This part of the study aims at discovering the interactions between opioids and other drugs that could lead to potential side effects in patients. In order to carry out this assessment, we considered the cohort of 749 patients who were diagnosed with side effects in the previous section (including all age groups). These patients were prescribed at least one of the 11 opioids under consideration and 3,710 other drugs put together. We categorized the side effects into seven groups and the summary is provided in Supplementary Table S1. All the opioids were assigned an index between 1 and 11 and the other drugs were also indexed in the same fashion. For each opioid and other drug combination, the number of patients who were diagnosed with side effects in each of the above seven groups were tabulated. These numbers are normalized between the range of 0 and 1 and then used for performing K-means clustering. From the elbow plot shown in Figure 5, the number of optimal clusters were found to be 4. Apart from the variants of regular salts like potassium chloride and sodium chloride, insulin is one important drug that has been classified into the predominant cluster. Additional information on this part of the study can be found in the Supplementary Material.

## Results

The results of this study can be summarized in three sections: (a) Predictive modelling for classifying patients susceptible to opioid abuse, (b) Predictive modelling for classifying patients susceptible to death and (c) Interactions between opioids and other drugs.

### Predictive Modeling for Classifying Patients Susceptible to Opioid Abuse

**FIGURE 6:**
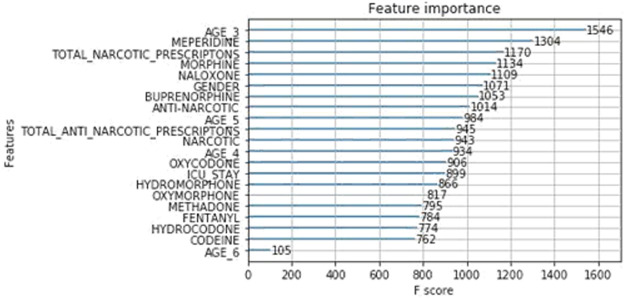
Importance of features.

As previously described, we implemented two models for classifying patients who may be prone to adverse events upon opioid consumption. Table 4 shows that XGBoost has outperformed the Logic Regression model. This could be due to the fact that each patient is associated with consumption of few opioids more than the others. And hence only a subset of features which are related to those particular opioids are more important than the others. Since XGBoost works by sub-sampling the features, the classification accuracy of the enhanced model is much higher than that of the baseline. From Figure 6, it can be observed that XGBoost has classified the AGE 3, a group with patients between 20 and 40 years of age, as the most important feature in deciding the patient’s susceptibility to adverse events, followed by the MEPERIDINE prescriptions and TOTAL NARCOTIC PRESCRIPTIONS. A more obvious result that follows our analysis of feature correlation is that NALOXONE and MORPHINE are also among the important contributing features.

**FIGURE 7:**
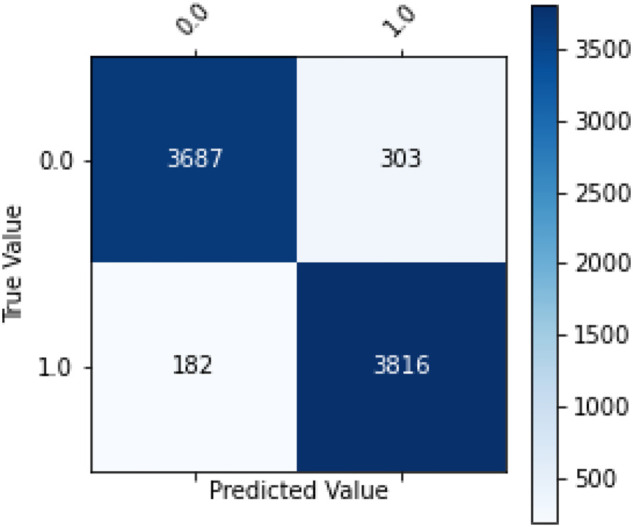
Confusion matrix.

Also, as hypothesized, XGBoost model is more sensitive in classifying patients with adverse events than those with no adverse events. Hence, the number of true positives for label 1 are more than those for label 0 (Figure 7). In other words, the model gave a better recall score than precision.

### Predictive Modeling for Classifying Mortality Risk

Unlike the previous model, it can be seen from Table 4 that the model used for classifying patients with high risk of mortality following opioid prescription has a higher precision score than the recall. This could be largely due to the small dataset (587) used for training the model.

### Adverse Event Risk due to Interactions Between Opioids and Other Drugs

As previously stated, insulin has been found to be in the predominant cluster associated with all categories of side effects. Not only has insulin been used widely in patients prescribed with opioids but the incidence of side effects has been comparatively large in the case of opioid and insulin combination. This observation backs up the results from two earlier studies conducted by Li et al., and Sharma et al. (Yu et al., 2003; Sharma and Balhara, 2016) The first study states that morphine could lead to desensitization of insulin receptor signaling. This could be one reason for increased usage of insulin in patients prescribed with opioids. The second study indicates that islet cells, which are responsible for the production of insulin, do not respond in an appropriate manner to the glucose signals in patients with opioid addiction.

## Discussion

While acknowledging the fact that the study done by Che et al., used a different dataset, it might be useful to have a glance over the performance in both the studies since the total number of patients experiencing opioid dependence and/or adverse effects in both the studies is same (Che et al., 2017). Our results show that the current models classify the patients with a better accuracy and recall by just using traditional machine learning models. Also, our enhanced model (94.35%) has better performance scores over the RNN model (76.07%) in Che et al., and can classify the patients irrespective of whether they are a short term or a long-term user (Che et al., 2017).

### Limitations of the Current Study

There are a few drawbacks associated with this study. The model for predicting mortality, unlike those for predicting the side effects, might not be robust since the reason for death of the patient remains undisclosed. Though the patient has experienced side effects, his/her death might not necessarily be related to opioid exposure. This analysis of mortality prediction should be considered as a preliminary step. Further, the study of interactions between opioids and other drugs is based solely on the frequency of prescription and the frequency of incidence of side effects. As we wanted to study the correlation between the incidence of side effects and the prescription opioids/drugs, irrespective of a patient’s characteristics, we didn’t include other interactions such as protein-protein, drug-target protein etc. like that in the study done by Zitnik et al. (2018).

## Conclusion

Opioids are a class of drugs used as pain relievers by binding to opioid receptors on nerve cells in the brain and the nervous system to mitigate pain. Addiction is one of the chronic and primary adverse events of prolonged usage of opioids. They may also cause psychological disorders, muscle pain, depression, anxiety attacks, etc. This study is intended to assist prescription of opioids. It aims at building a predictive model to classify the patients of interest into two categories based on their susceptibility to opioid abuse. We trained two classification models, Logistic Regression with L2 regularization (baseline) and Extreme Gradient Boosting (enhanced model), to achieve this task. These results suggest that the enhanced model provides a promising approach to identify patients who are most vulnerable to adverse events when given opioids. If employed as a reassurance technique, this study could be of tremendous help to medical practitioners in designing an appropriate action plan for their patients before prescribing them opioids and will help combat the opioid epidemic.

## Data Availability

The original contributions presented in the study are included in the article/Supplementary Material, further inquiries can be directed to the corresponding authors.

## References

[B1] Averill L. A. Averill C. L. Lyndsay A Staley J. L. O-K. Kauwe John. S. K. Henrie-Barrus P. (2017). The Opioid Abuse Risk Screener Predicts Aberrant Same-Day Urine Drug Tests and 1-year Controlled Substance Database Checks: A Brief Report. Health Psychol. Open 4 (2), 2055102917748459. 10.1177/2055102917748459 29379630 PMC5779942

[B2] Bartoli A. Kominek C. (2019). What Do the CDC Guidelines Mean for Patients on Long-Term, High-Dose Opioids? Practical Pain Management. Second Edition.

[B3] Bowyer K. W. Chawla N. V. Hall L. O. Philip Kegelmeyer W. (2011). SMOTE: Synthetic Minority Over-sampling Technique. CoRR, abs/ 11061813.

[B4] Butler S. F. Budman S. H. Fernandez K. C. Fanciullo G. J. Jamison R N. (2009). Cross-validation of a Screener to Predict Opioid Misuse in Chronic Pain Patients (SOAPP-R). J. Addict. Med. 3, 66–73. 2009 2009. Copyright - c 2009, American Society of Addiction Medicine; Date completed - 2008-09-17; Date created - 2008-06-30; Date revised - 20091214; Number of references - 24; Last updated - 2016-11-18; SubjectsTermNotLitGenreText - Chronic Pain 1490 1493 6060 8453 8698 ; Opiates 2599 5524 5940 8698 ; Psychometrics 5061 6956 8698 ; Screening 5061 7601 8698 ; Test Validity 5061 8609 8625 8630 8698 ; 6216 8698 ; 5061 8609 8618 8630 8698. 10.1097/adm.0b013e31818e41da 20161199 PMC2712300

[B5] Butler S. Fernandez K. Benoit C. Simon B. Jamison R. (2008). Validation of the Revised Screener and Opioid Assessment for Patients with Pain (Soapp-r). J. pain : official J. Am. Pain Soc. 9, 360. 10.1016/j.jpain.2007.11.014 PMC2359825 18203666

[B6] Calcaterra S. L. Scarbro S. Hull M. L. Forber A. D. Binswanger I. A. Colborn K. L. (2018). Prediction of Future Chronic Opioid Use Among Hospitalized Patients. J. Gen. Intern. Med. 33 (6), 898–905. 10.1007/s11606-018-4335-8 29404943 PMC5975151

[B7] Che Z. St Sauver J. Liu H. Liu Y. (2017). Deep Learning Solutions for Classifying Patients on Opioid Use. AMIA Annu. Symp. Proc. AMIA Symp, 525–534. 29854117 PMC5977635

[B8] Chen T. Guestrin C. (2016). “Xgboost: A Scalable Tree Boosting System,” in Proceedings of the 22nd ACM SIGKDD International Conference on Knowledge Discovery and Data Mining - KDD ’16, 785–794. Available at: https://app.dimensions.ai.on.2018/12/08 .

[B9] Chiu A. S. Jean R. A. Hoag J. R. Freedman-Weiss M. Healy J. M. Pei K. Y. (2018). Association of Lowering Default Pill Counts in Electronic Medical Record Systems with Postoperative Opioid Prescribing. JAMA Surg. 153 (11), 1012–1019. 10.1001/jamasurg.2018.2083 30027289 PMC6583068

[B10] Dowell D. Haegerich T. M. Chou R. (2016). CDC Guideline for Prescribing Opioids for Chronic Pain^aunited States. JAMA 315 (15), 1624–1645. 10.1001/jama.2016.1464 26977696 PMC6390846

[B11] Hall A. J. Logan J. E. Toblin R. L. Kaplan J. A. Kraner J. C. Bixler D. (2008). Patterns of Abuse Among Unintentional Pharmaceutical Overdose Fatalities. JAMA 300 (22), 2613–2620. 10.1001/jama.2008.802 19066381

[B12] Heslin K. C. Elixhauser A. Steiner C. A. (2015). ICD-9 CM Diagnosis Codes Defining Substance Use Disorders (Rockville (MD): Agency for Healthcare Research and Quality (US)). pages Table 4.Hospitalizations Involving Mental and Substance Use Disorders Among Adults, 2012: Statistical Brief 191. Healthcare Cost and Utilization Project (HCUP) Statistical Briefs [Internet]

[B13] Jeffery M. M. Hooten W. M. Henk H. J. Fernanda Bellolio M. Hess E. P. Meara E. (2018). Trends in Opioid Use in Commercially Insured and Medicare Advantage Populations in 2007-16: Retrospective Cohort Study. BMJ 362. 10.1136/bmj.k2833 PMC6066997 30068513

[B14] Gong J. J. Jacobs A. Z. Stuart T. E. de Vaan M. Discovering Heterogeneous Subpopulations for fine-grained Analysis of Opioid Use and Opioid Use Disorders. 11 2018.

[B15] Koob G. F. Ma Arends. Moal M. L. (2014). Drugs, Addiction, and the Brain. chapter 4 - psychostimulants. Cambridge, Massachusetts: Academic Press, 93–132. 10.1016/b978-0-12-386937-1.00004-0

[B16] Koob G. F. Moal M. L. (2006). Neurobiology of Addiction. chapter 3 - psychostimulants. Cambridge, Massachusetts: Academic Press, 69–120.

[B17] Kringel D. Ultsch A. Zimmermann M. Jansen J-P. Ilias W. Freynhagen R. (2016). Emergent Biomarker Derived from Next Generation Sequencing to Identify Pain Patients Requiring Uncommonly High Opioid Doses. Pharmacogenomics J. 17, 05. 10.1038/tpj.2016.28 PMC5637232 27139154

[B18] Latif Z.-H. Benth J. Ś Solli K. K. Opheim A. Kunoe N. Krajci P. (2018). Anxiety, Depression, and Insomnia Among Adults with Opioid Dependence Treated with Extended-Release Naltrexone vs Buprenorphine-Naloxone: A Randomized Clinical Trial and Follow-Up Study. JAMA Psychiatry 76(2):127-134. 10.1001/jamapsychiatry.2018.3537 PMC6439739 30566177

[B19] Miotto R. Wang Fei. Wang S. Jiang X. Dudley J. T. (2017). Deep Learning for Healthcare: Review, Opportunities and Challenges. Brief. Bioinformatics 19 (6), 1236–1246. 10.1093/bib/bbx044 PMC6455466 28481991

[B20] Moore B. J. Barrett M. L. (2017). Appendix A. ICD-9 CM and ICD-10 CM Opioid Related Diagnosis Codes Used in This Study. Rockville, Maryland: U.S. Agency for Healthcare Research and Quality.Case Study: Exploring How Opioid-Related Diagnosis Codes Translate from Icd-9-Cm to Icd-10-Cm. Online

[B21] Rudd R. A. Seth P. David F. Scholl L. (2016). Increases in Drug and Opioid-Involved Overdose Deaths - united states, 2010-2015. MMWR Morb. Mortal. Wkly. Rep. 65, 1445–1452. 10.15585/mmwr.mm655051e1 28033313

[B22] Shameer K. Badgeley M. A. Miotto R. Glicksberg B. S. Morgan J. W. Dudley J. T. (2017). “Translational Bioinformatics in the Era of Real-Time Biomedical, Health Care and Wellness Data Streams,” in Briefings in Bioinformatics. 10.1093/bib/bbv118 PMC5221424 26876889

[B23] Shameer K. Johnson K. W. Glicksberg B. S. Dudley J. T. Sengupta P. P. (2018). The Whole Is Greater Than the Sum of its Parts: Combining Classical Statistical and Machine Intelligence Methods in Medicine. Heart 104 (14), 1228. 10.1136/heartjnl-2018-313377 29945951

[B24] Sharma P. Balhara Y. (2016). Opioid Use and Diabetes: An Overview. J. Soc. Health Diabetes 4 (1)006-010. 10.4103/2321-0656.176570

[B25] Steinman M. A. Landefeld C. (2018). Overcoming Inertia to Improve Medication Use and Deprescribing. JAMA 320 (18), 1867–1869. 10.1001/jama.2018.16473 30422182 PMC6342186

[B26] Tyndale R. Sellers E. (2018). Opioids: The Painful Public Health Reality. Clin. Pharmacol. Ther. 103, 924–935. 10.1002/cpt.1074 29878319

[B27] Wiese H. J. Piercey R. Clark C. D. (2018). Changing Prescribing Behavior in the united states: Moving Upstream in Opioid Prescription Education. Clin. Pharmacol. Ther. 103 6, 982–989. 10.1002/cpt.1015 29315508

[B28] Wong A. Plasek J. M. Montecalvo S. P. Zhou Li. (2018). Natural Language Processing and its Implications for the Future of Medication Safety: A Narrative Review of Recent Advances and Challenges. Pharmacother. J. Hum. Pharmacol. Drug Ther. 38. 10.1002/phar.2151 29884988

[B29] Yu L. Eitan S. Wu J. Evans C. Kieffer B. Sun X. (2003). Morphine Induces Desensitization of Insulin Receptor Signaling. Mol. Cell. Biol. 23, 6255–6266. 10.1128/mcb.23.17.6255-6266.2003 12917346 PMC180943

[B30] Zitnik M. Agrawal M. Leskovec J. (2018). Modeling Polypharmacy Side Effects with Graph Convolutional Networks. Bioinformatics 34, i457–i466. 29949996 10.1093/bioinformatics/bty294 PMC6022705

